# Virological and Serological Characterisation of SARS-CoV-2 Infections Diagnosed After mRNA BNT162b2 Vaccination Between December 2020 and March 2021

**DOI:** 10.3389/fmed.2021.815870

**Published:** 2022-01-20

**Authors:** Francesca Colavita, Silvia Meschi, Cesare Ernesto Maria Gruber, Martina Rueca, Francesco Vairo, Giulia Matusali, Daniele Lapa, Emanuela Giombini, Gabriella De Carli, Martina Spaziante, Francesco Messina, Giulia Bonfiglio, Fabrizio Carletti, Eleonora Lalle, Lavinia Fabeni, Giulia Berno, Vincenzo Puro, Barbara Bartolini, Antonino Di Caro, Giuseppe Ippolito, Maria Rosaria Capobianchi, Concetta Castilletti

**Affiliations:** ^1^National Institute for Infectious Diseases “Lazzaro Spallanzani” IRCCS, Roma, Italy; ^2^Unicamillus, International Medical University, Roma, Italy

**Keywords:** COVID-19, SARS-CoV-2, viral variants, neutralising antibodies, vaccine, breakthrough infection, Italy

## Abstract

**Background:**

Vaccines for coronavirus disease 2019 (COVID-19) are proving to be very effective in preventing severe illness; however, although rare, post-vaccine infections have been reported. The present study focuses on virological and serological features of 94 infections that occurred in Lazio Region (Central Italy) between 27 December 2020, and 30 March 2021, after one or two doses of mRNA BNT162b2 vaccine.

**Methods:**

We evaluated clinical features, virological (viral load; viral infectiousness; genomic characterisation), and serological (anti-nucleoprotein Ig; anti-Spike RBD IgG; neutralising antibodies, nAb) characteristics of 94 post-vaccine infections at the time of diagnosis. Nasopharyngeal swabs (NPSs) and serum samples were collected in the framework of the surveillance activities on SARS-CoV-2 variants established in Lazio Region (Central Italy) and analysed at the National Institute for Infectious Diseases “L. Spallanzani” in Rome.

**Results:**

The majority (92.6%) of the post-vaccine infections showed pauci/asymptomatic or mild clinical course, with symptoms and hospitalisation rate significantly less frequent in patients infected after full vaccination course as compared to patients who received a single dose vaccine. Although differences were not statistically significant, viral loads and isolation rates were lower in NPSs from patients infected after receiving two vaccine doses as compared to patients with one dose. Most cases (84%) had nAb in serum at the time of infection diagnosis, which is a sub-group of vaccinees, were found similarly able to neutralise Alpha and Gamma variants. Asymptomatic individuals showed higher nAb titres as compared to symptomatic cases (median titre: 1:120 vs. 1:40, respectively). Finally, the proportion of post-vaccine infections attributed either to Alpha and Gamma variants was similar to the proportion observed in the contemporary unvaccinated population in the Lazio region, and mutational analysis did not reveal enrichment of a defined set of Spike protein substitutions depending on the vaccination status.

**Conclusion:**

Our study conducted using real-life data, emphasised the importance of monitoring vaccine breakthrough infections, through the characterisation of virological, immunological, and clinical features associated with these events, in order to tune prevention measures in the next phase of the COVID-19 pandemic.

## Introduction

In <12 months after the beginning of the coronavirus disease 2019 (COVID-19) pandemic, scientific research succeeded in developing multiple vaccines against a previously unknown viral pathogen, severe acute respiratory coronavirus 2 (SARS-CoV-2). The mRNA-based Pfizer-BioNTech vaccine (BNT162b2) has been the first authorised, and on 27 December 2020, the European Union countries launched a coordinated vaccination campaign that initially was prioritised for individuals at high risk of SARS-CoV-2 exposure, such as the healthcare workers (HCW), and those at high risk of severe COVID-19, including elderly and residents of assisted living facilities. Its effectiveness in preventing severe diseases and death is well documented together with the impact in reducing the overall transmission rate of SARS-CoV-2 (1–3). However, COVID-19 vaccines do not offer 100% protection against SARS-CoV-2 infection, and breakthrough infections can occur in the vaccinated population (4, 5). Virological and immunological investigation on these cases is crucial to better characterise features of breakthrough infections and their impact on the pandemic. The emergence of SARS-CoV-2 variants in the Spike protein represents one of the concerns for vaccine effectiveness; in fact, some showed the potential of immunological escape from the antibodies response potentially leading to COVID-19 epidemic rebounds (6–8). Here, we described virological and serological testing performed at the Regional Reference Laboratory (RRL) of Virology of the National Institute for Infectious Diseases “L. Spallanzani” (INMI) in Rome, Italy, on samples collected at the time of diagnosis from 94 individuals, who resulted positive for SARS-CoV-2 between 27 December 2020 and 30 March 2021, following one or two doses of BNT162b2 vaccine. This study was conducted in the framework of specific surveillance on SARS-CoV-2 variants established in the Lazio Region (Central Italy) aimed to identify the circulation of variants associated with vaccine escape in the general population.

## Methods

### Study Group

In the frame of the Regional Surveillance programme, NPSs and, possibly, sera collected from individuals who resulted positive for SARS-CoV-2 after vaccination were sent to INMI in Rome, Italy, for further laboratory investigation. These individuals were tested at peripheral laboratories either following symptoms onset, for contact tracing, or screening activities. In this study, we included the first batch of samples (94 NPSs) which were referred between 27 December 2020 and 30 March 2021 to the RRL for virological evaluation of post-vaccination RT-PCR positivity occurred at least 1 day after one or two BNT162b2 vaccine doses. Reporting of the clinical course was based on the COVID-19 integrated national surveillance system (source: https://www.epicentro.iss.it/coronavirus/bollettino/Bollettino-sorveglianza-integrata-COVID-19_10-novembre-2021.pdf;last access: 15/11/2021). For 79 individuals, a known date of vaccination was reported and the time lapsing from vaccination to sample collection was calculated. We classified the individuals with known vaccination dates into three groups based on the time elapsed from the first dose of vaccine to infection, i.e., time of SARS-CoV-2 positive test or symptoms onset (here considered both as infection starting date): Group 1, individuals tested positive 1–15 days after the first dose; Group 2, 16–30 days after first dose vaccination; Group 3, >30 days from first dose (10 days after the second dose injection, considered a full vaccinated group). For 50 individuals (44 of them with the reported date of vaccination), a serum sample collected at the time of diagnosis was also available for serological testing. In fact, according to the local surveillance system, serum collection was recommended but not mandatory for post-vaccine infections follow-up. NPSs and serum samples were shipped to INMI under controlled temperature (−80°C and refrigerated at +4°C, respectively). Sequencing data (*n* = 1,072) produced at INMI exclusively from randomly selected samples collected from unvaccinated individuals during the same study period and representing all the regional territory, were used to evaluate the prevalence of variants.

### Molecular Testing and Virus Characterisation

Semi-quantitative estimation of viral load was assessed by RT-PCR using DiaSorin Simplexa^®^ COVID-19 Direct kit (DiaSorin, Saluggia, Italy). For whole-genome sequencing, Next-Generation Sequencing (NGS) was carried out on Ion Torrent or Illumina Platform using Ion AmpliSeq SARS-CoV-2 Research Panel, following manufacturer's instructions (ThermoFisher, USA). Complete genome sequences were obtained combining in-house pipeline ESCA (9) with IRMA (10) and DRAGEN RNA Pathogen Detection 3.5.15 (Illumina BaseSpace, Illumina, USA) software and submitted on the GISAID platform (11). In case of low coverage for the full-genome characterisation, Sanger sequencing was used to fill the NGS gaps in the Spike coding gene.

### Virus Isolation

The viral culture was performed in a biosafety level 3 (BSL-3) laboratory at INMI on Vero E6/TMPRSS2 (kindly provided by Dr. Oeda S., National Institute of Infectious Diseases, Tokyo, Japan), as previously described (12). NPSs were stored at −80°C after collection and seeded on cells immediately after a single thawing.

### Serological Testing

Anti-N and anti-RBD Spike IgG were evaluated using Abbott SARS-CoV-2 assay on Abbott ARCHITECT^®^ i2000sr (Abbott Diagnostics, Chicago, IL, USA) and neutralising antibodies (nAb) titres measured using SARS-CoV-2 microneutralisation test (MNT) based on the live virus (13). The viral strains used in MNT were: (i) B.1—clade G (GISAID accession number: EPI_ISL_568579, EVAG Ref-SKU: 008V-04005); (ii) B.1.1.7—clade GRY, alpha variant (GISAID accession number: EPI_ISL_913449, EVAG Ref-SKU: 008V-04050); iii) P.1—clade GR/501Y.V3, Gamma variant (GISAID accession number: EPI_ISL_1290803, EVAG Ref-SKU: 008V-04101). SARS-CoV-2 neutralisation titres were expressed as the reciprocal of the highest serum dilution inhibiting at least 90% of the cytopathic effect.

### Statistics

Epidemiological and demographic data were extracted from the Regional Surveillance Information System and analysed using the STATA 14 software (StataCorp LLC, USA). Demographic characteristics of the vaccinated individuals were described using median and interquartile range (IQR) for continuous parameters, and absolute and relative (percentage) frequencies for categorical variables. Inferential analysis of association was performed using chi-square or Fisher exact tests for categorical variables, and Mann–Whitney or Kruskal-Wallis tests for continuous parameters. When comparing neutralisation titre against different variants, the Friedman-Dunn test was used. Univariate analysis and odds ratio (OR) with 95% CI were shown. Analyses were performed using GraphPad Prism version 8 (GraphPad Software, La Jolla California, USA) and SPSS 23 (IBM, USA) for Windows statistical software; *p* < 0.05 was considered statistically significant.

### Ethics

This work was performed within the framework of the COVID-19 outbreak response and surveillance program and the laboratory characterisation of post-vaccination infections by the INMI laboratory as the RRL is an essential part of the Lazio surveillance regional plan. The use of laboratory and epidemiological records for research purposes has been approved by the INMI Ethical Committee (issue n. 214/20-11-2020), and the need for an informed consent form was waived. The study has been conducted in respect of current legislation on personal data protection, all data are presented in non-identifiable form.

## Results

### Cases of SARS-CoV-2 Infections After BNT162b2 Vaccination

According to the Regional Surveillance Information System, from 27 December 2020, the start of the vaccination campaign, up to 30 March 2021, 130,761 SARS-CoV-2 cases were reported in the Lazio region, the majority (126,670, 96.4%) were unvaccinated. Among the 735,616 individuals who received one or two doses of the BNT162b2 vaccine in the same period in the Lazio region, 1,879 (0.26%) tested positive for SARS-CoV-2 at least 1 day after vaccination; the majority (79.5%) of these individuals did not complete the full vaccination course. This study described the results obtained on the first 94 NPSs of post-vaccine infected individuals diagnosed at peripheral laboratories and sent to the RRL in the framework of the COVID-19 outbreak response and surveillance regional program. The median time between infection recognition (as symptoms onset or time of the first diagnosis for asymptomatic patients) and testing was 0.5 days (range: 0–9 days, with 7 tested samples collected >4 days after diagnosis or symptoms onset). Demographic and epidemiologic data, including clinical information, are shown in Table 1. None of the cases reported previous SARS-CoV-2 infection. The majority (*n* = 82, 87.2%) of the 94 individuals under investigation were HCW, the remaining samples were from elderly people (over 80 years old). The median age was 50.5 years old (IQR: 62–39.8), 56 (59.6%) were women. Moreover, 49 (52.1%) were asymptomatic at the diagnosis and underwent SARS-CoV-2 testing for periodic screening or as contacts of positive cases. The majority (*n* = 61, 64.9%) of post-vaccination cases had pauci/asymptomatic clinical course, while a mild disease was reported for 26 (27.7%); severe illness was reported for 7 (7.4%) patients, all with one or more pre-existing co-morbidities, including cardiovascular chronic diseases, diabetes, obesity, renal affections, and neurological disorders, 4 were over 80 years old. Age and co-morbidities were significantly associated with severe disease (*p* = 0.008 and <0.001, respectively). According to the information available at the time of writing (*n* = 85), most infected persons (97.6%) cleared the virus and recovered, while 2 patients died; both dead patients were over 85 years old, presented pre-existing co-morbidities (i.e., cardiovascular chronic diseases, diabetes, and neurological disorders), and tested positive after full vaccination (9 and 19 days after the second dose, respectively). For 79 individuals, a known date of vaccination was reported (Table 1). The median time between the first-dose vaccination and symptoms onset, or time of the first diagnosis for asymptomatic cases, was 47 days, ranging from 1 to 85 days after the first dose (corresponding to 64 days following the full vaccination). Furthermore, 54 (68.4%) individuals resulted infected after full vaccination. Amongst these cases, the median time between vaccination and infection diagnosis was 48.5 (IQR 36–67.5) days for HCW vs. 31 (IQR 28–38.5) days for elderly over 80 years old (*p* = 0.184). Symptoms at diagnosis and hospitalisation rate were significantly less frequent in patients infected after full vaccination course as compared to patients infected after a single dose; a trend towards less frequent severe course was observed in infections acquired after two doses.

**Table 1:** Demographic and epidemiological information is available for the study cohort.

**Characteristic**	**Total cases tested positive after vaccination (*N =* 94)**	**Cases with known date of vaccination (*N =* 79)**			
		**Group 1 (*n =* 9, 11.4%)**	**Group 2 (*n =* 16, 20.3%)**	**Group 3 (*n =* 54, 68.4%)**	***p*-value^a^**
**Age** (median, range)	55.5 (23-92)	53 (24-59)	57.5 (24-82)	49 (23-87)	0.540
**Gender (*****n*****, %)** Male	38 (40.4%)	2 (22.2%)	8 (50.0%)	22 (40.7%)	0.397
Fe4	56 (59.6%)	7 (77.8%)	8 (50.0%)	32 (59.3%)	
**Category (*****n*****, %)** HCW	82 (87.2%)	9 (100.0%)	13 (81.3%)	50 (92.6%)	0.228
Age over 80	12 (12.8%)	–	3 (18.8%)	4 (7.4%)	
**Symptoms at diagnosis (** ***n*** **, %)**
Yes	45 (47.9%)	8 (88.9%)	8 (50.0%)	23 (42.6%)	**0.037**
No	49 (52.1%)	1 (11.1%)	8 (50.0%)	31 (57.4%)	
**Clinical course (** ***n*** **, %)**
Pauci/asymptomatic	61 (64.9%)	5 (55.5%)	10 (62.5%)	38 (70.4%)	0.208
Mild	26 (27.7%)	2 (22.2%)	6 (37.5%)	13 (24.1%)	
Severe	7 (7.4%)	2 (22.2%)	–	3 (5.5%)	
**Q18Hospitalisation (** ***n*** **, %)**
Yes	10 (10.6%)	2 (22.2%)	1 (6.3%)	4 (7.4%)	**0.046**
Admission to ICU	1 (10.0%)	–	–	–	
No	84 (89.4%)	7 (77.8%)	15 (93.7%)	50 (92.6%)	
**COVID-19 outcome (** ***n*** **, %)** ^#^
Recovered	83 (97.6%)	9 (100.0%)	15 (100.0%)	46 (95.8%)	0.598
Dead	2 (2.4%)	-	-	2 (4.2%)	

a *By Kruskal-Wallis tests (for quantitative variables) and Chi-squared test (for categorical variables)*.

*p-values < 0.05 were considered statistically significant (in bold)*.

# *Available at the time of analysis for the following: total vaccinees, 85/94; Group 1, 9/9; Group 2, 15/16; Group 3, 48/54*.

### SARS-CoV-2 Viral Loads and Infectivity in NPSs From Individuals Infected After Vaccination

**Figure 1:**
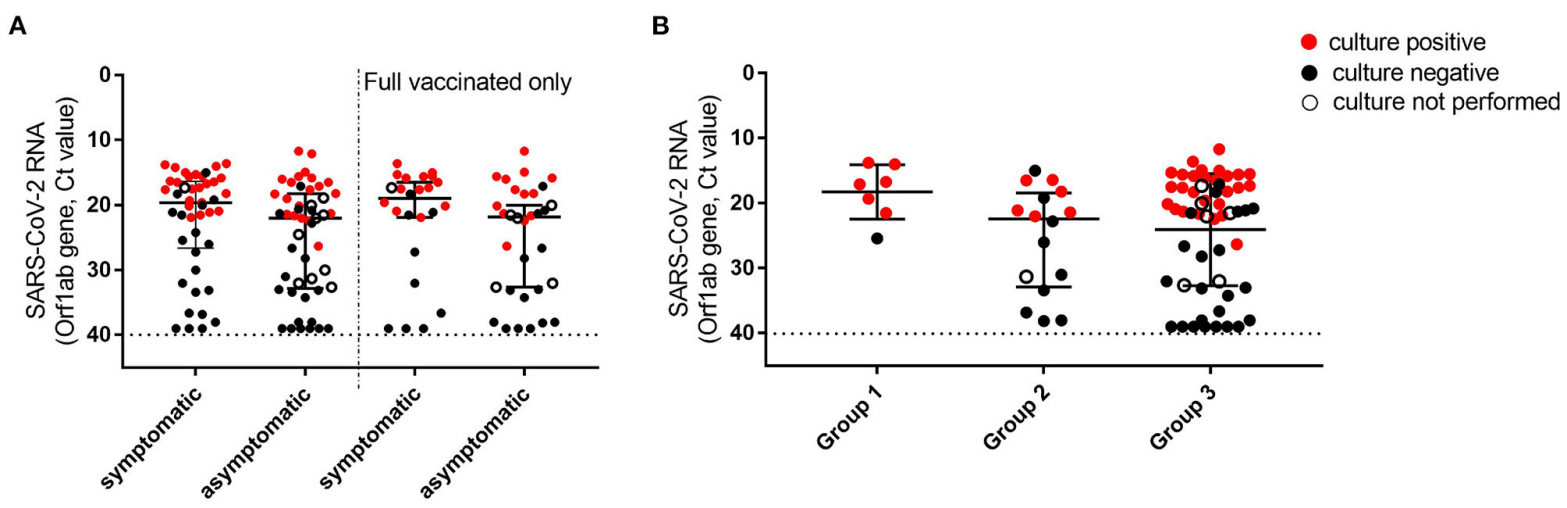
Viral loads and viral culture outcomes in NPSs collected in individuals tested positive after first dose vaccination. **(A)** Viral loads were detected in all symptomatic and asymptomatic individuals at the time of diagnosis (left, *n* = 45 and 49, respectively), and in a subgroup (Group 3) including only individuals who tested positive after 10 days from second dose vaccination (right, *n* = 24 and 32, respectively). **(B)** Viral loads detected in NPSs were collected at different time points from the first dose vaccination. Group 1 (time lapse 1–15 days), *n* = 7; Group 2 (16–30 days), *n* = 16; Group 3 (>30 days), *n* = 56. Viral RNA levels are expressed as Ct of Orf1ab gene amplification, the horizontal dashed line represents the limit of detection of RT-PCR (Ct: 40). Median Ct values and IQR are shown. Statistical analysis was performed in **(A)** by Mann–Whitney test, *p* = 0.053 (left) and *p* = 0.098 (right); in **(B)** by Kruskal-Wallis test, *p* = 0.135. Samples yielding positive or negative viral culture are indicated in red and black, respectively; empty dots indicate samples for which viral culture was not performed.

Median Ct values of NPSs collected from vaccinees at diagnosis was 21.2 (IQR: 17.5–31.3), with no significant difference between asymptomatic and symptomatic patients (median Ct values: 22 vs. 19.6, Figure 1A). The Ct values were similar to those detected at the time of infection diagnosis in a group of unvaccinated individuals, matched for gender and age, presenting at INM with mild symptoms between 1st of January and 30th of March 2021 (*n* = 31, median Ct value: 19.4, IQR: 18–28.7; *p* = 0.204). The proportion of samples with Ct >30 in asymptomatic individuals (32.6%) was higher compared to the symptomatic patients (20.0%) but did not reach statistical significance (*p* = 0.242). Ct values resulted in similar also in all 3 groups identifying patients according to the time elapsed from the first dose to diagnosis (known for 79 individuals), despite the higher number of samples with Ct>30 detected in Group 3 (Figure 1B). To understand whether viral RNA was associated with infectiousness, virus isolation was attempted on 84 NPSs; 10 NPSs were not tested due to viral infectivity inactivation by guanidine isothiocyanate contained in the transport medium used for sample collection. Notably, infectious virus was rescued from 44 (52.4%) NPSs, 24 (54.5%) collected from symptomatic individuals, 20 (50%) collected from asymptomatic subjects (*p* = 0.827); similar results were obtained when considering fully vaccinated patients only (60.9% in symptomatic vs. 48.2% in asymptomatic, *p* = 0.567). As shown in detail in Supplementary Figure S1A, over 39 positive viral cultures, 27 (67.5%) were obtained from fully vaccinated individuals, up to 85 days after the first vaccination dose. The isolation rate according to the time elapsed from the first dose to diagnosis was found higher in samples collected shortly after the vaccination (Group 1) but did not reach a statistical difference compared to the other groups (Group 1: 85.7%, Group 2: 40%; Group 3: 54%, *p* = 0.411). Overall, the median Ct value of the samples with positive viral culture was 17.5 (IQR 15.6–20.1), and isolation of the infectious virus was strongly associated only with the viral RNA load in the NPSs, with OR >100 for Ct ≤ 25 vs. Ct > 25 (*p* < 0.001, Supplementary Table S1).

### Antibody Response at the Time of Infection Diagnosis

Serological testing was performed on the available serum samples (*n* = 50) which have been collected at the time of diagnosis at the peripheral laboratories and sent to the RRL (Supplementary Table S2). The results showed that antibody response at the time of infection diagnosis was detected in 48 individuals (96%, median anti-RBD Spike IgG BAU/ml = 704.6, IQR 403–2,111); 42 (84%) of them presented also detectable nAb (median titre = 1:80, IQR 1:40–1:160), mostly (66.7%) fully vaccinated (Supplementary Figure S1B). Furthermore, eight vaccinees (16%) did not show detectable nAb at diagnosis, of whom 2 (4%) were fully vaccinated (both over 80 years old and presenting co-morbidities), indicating primary non-response to the vaccine. Anti-N IgG at diagnosis was observed only in 3 patients. Notably, we found higher nAb levels in asymptomatic individuals (median titre: 1:120, IQR 96.2–361.9) at the diagnosis as compared to symptomatic cases (median titre: 1:40, IQR 42.1–274.1). In addition, we assessed the possible association between nAb titres and disease severity. We found positive OR but not statistically significant association between low nAb titre and worse clinical course (OR 4.263, 95% CI: 0.411–44.169; *p* = 0.224), or presence of symptoms at diagnosis (OR 2.232, 95% CI: 0.714–6.973; *p* = 0.167), or hospitalisation (OR 6, 95% CI: 0.619–58.135; *p* = 0.122). On the other hand, although a trend to negative association of viral isolation rate with high nAb titres was observed (OR = 0.28 for nAb ≥1:80 vs. <1:80, *p* = 0.054, Supplementary Supplementary Table S1), the presence of nAb in serum did not preclude virus isolation from NPSs, and titres were not correlated with viral load (median Ct values: 21.5, IQR 16.5–32.2; Spearman *r* = 0.22, *p* = 0.124). Functional humoral response detected at the time of diagnosis in a sub-group of cases (*n* = 18) was effective also against the variants causing the infection. Indeed, no significant differences in nAb titres were observed against B.1.177, Alpha, and Gamma variants (*p* = 0.656) (Supplementary Table S3).

### Distribution of SARS-CoV-2 Variants in Post-Vaccination Infections

**Figure 2:**
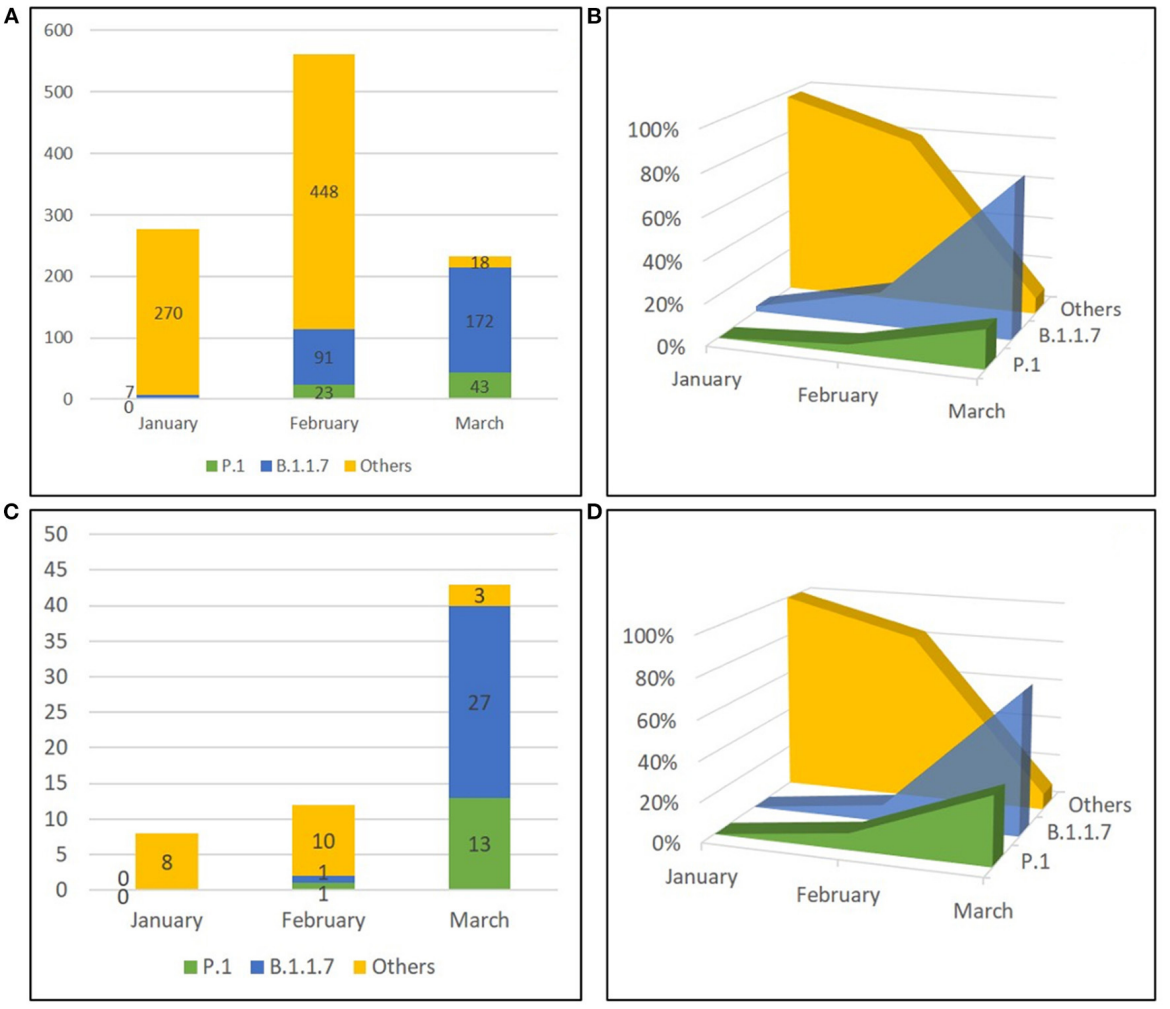
Temporal distribution of PANGO lineages of SARS-CoV-2 genomes. Sequences obtained from unvaccinated individuals **(A,B)** and from vaccinated subjects **(C,D)** in Lazio Region (Central Italy) between January and March 2021 are shown as absolute frequencies **(A, C)** and percentages **(B,D)**. Others included strains belonging to B.1.177 lineage, B.1.525 lineage, B.1.1 lineage, B.1.1.39 lineage, B.1 lineage, and B.1.258.17 lineage.

We next investigated the viral variants infecting the vaccinated individuals included in this study. Whole-genome sequences (WGS) were obtained from the 58 NPS samples; additional variant strain identification was obtained for 5 patients by partial Sanger sequencing of the S region. Out of these 63 SARS-CoV-2 sequences, 15 (23.8%) belonged to B.1.177 lineage (GV clade), 14 (22.2%) were Gamma variants (P.1 lineage, GR clade) and 28 (44.4 %) were Alpha variants (B.1.1.7 lineage, GRY clade). One (1.6%) resulted to be Variant of Interest (VOI) Eta (B.1.525 lineage, G clade), and 5 (7.9%) sequences belonged to other clades with 3 sequences to the clade GR (i.e., 2 of the B.1.1 lineage, 1 of the B.1.1.39 lineage) and 2 to the clade G (i.e., 1 of the B.1 lineage and 1 of the B.1.258.17 lineage) (Supplementary Figure S2A). The distribution of SARS-CoV-2 variants amongst the vaccinated individuals grouped according to vaccination status (i.e., days from vaccination) was related to the time of infection diagnosis and reflected mostly the circulation of the variants in the general population at the time of the infection Supplementary Figure S2B. For instance, B.1.1.7 was detected mainly in those vaccinated individuals who tested positive in March 2021, regardless of the vaccination status; accordingly, Group 3 showed the predominance of B.1.1.7 as this group included mainly vaccinated individuals who tested positive in March 2021. As matter of fact, when we compared our study population to an unvaccinated contemporary population, based on 1,072 samples randomly collected from Lazio patients between January and March 2021 and sequenced by NGS and Sanger for surveillance purposes, we observed that variants prevalence in vaccinated individuals followed the circulation in the general population. As shown in Supplementary Figure S3, we observed a similar temporal distribution between the two populations, with a clear increase of B.1.1.7 followed by P.1 in both groups, without significant difference for both P.1 (*p* = 0.08 in Chi-square test) and B.1.1.7 variants (*p* = 0.2 in Chi-square test) (Figure 2). Mutational analysis of Spike protein sequences obtained by NGS showed that signature mutations for the detected variants are observed in all groups, and other changes are found in a minority of patients (Supplementary Figure S3); especially for P.1, none of these minor changes seems to be enriched in Group 3, suggesting no association with more resistant forms.

## Discussion

The present study investigated 94 infections evaluated at the time of diagnosis, occurring in Lazio Region (Central Italy) after first or second dose administration of mRNA BNT162b2 vaccine, both from the host side (patients' demographics, infection severity, antibody status) and from the virus side (viral load, infectivity, and infecting variants).

Case-control and population-level studies widely demonstrated that vaccination substantially reduces both asymptomatic and symptomatic infections, and significantly prevents severe COVID-19 (2, 14–17). However vaccines are not sterilising and SARS-CoV-2 infections in previously vaccinated individuals can occur, although more likely to have a favourable outcome (18–20), even for immune-compromised individuals who were reported among the highest risk groups in experiencing breakthrough infections (21). Notably, breakthrough infections after vaccination against SARS-CoV-2 are increasingly reported, possibly due to the combined effect of the waning of vaccine-induced antibody levels and the emergence of new variants (22).

During the study period (27 December 2020–March 2021), a very tiny proportion (0.26%) of individuals was reported infected after vaccination over the total individuals who received the vaccine in the Lazio region. In addition, over 130,761 SARS-CoV-2 cases were reported during the same period in the Regional Surveillance Information System, only 2.45% of infections were in fully vaccinated people. Our study confirmed that the majority of infections observed in the analysed vaccinated individuals had a pauci/asymptomatic or mild clinical course. Severe disease was significantly less frequent in patients infected after a full vaccination course as compared to the patients who acquired the infection after a single dose, supporting the importance of the full vaccination schedule in naïve individuals. Those patients with severe symptoms presented pre-existing co-morbidities (i.e., cardiovascular chronic diseases, diabetes, obesity, renal affections, and neurological disorders) and those aged over 80, which represent still relevant risk factors for disease severity and are prognostic for a negative outcome (3, 23–27).

Neutralising antibodies (nAb) are generally detectable within 7–15 days of disease onset in individuals infected with SARS-CoV-2 (24). We found that the majority (84%) of the individuals who tested positive after vaccination had nAb at the initial time of the infection, 64.3% of them, fully vaccinated, showing high nAb titres (≥1:80). Bergwerk et al. (28) reported that nAb titres in infected vaccinees detected within a week before SARS-CoV-2 diagnosis were lower than those detected in uninfected vaccinees, and higher peri-infection nAb titres were associated with lower infectivity (higher Ct values). In our investigation, antibody levels prior to infection were not available for most of these individuals (except for two cases, who underwent serological testing 38 and 60 days before the infection diagnosis: nAb titres of 1:160 at day 15 from full immunisation for both). In line with recent reports (19, 20, 25), the nAb levels measured shortly after the infection, together with negative anti-N IgG for most cases, suggest that these functional immune responses may be the consequence of vaccination, not sufficient to prevent the contagion, but likely involved in protecting from symptomatic infection (29, 30). Indeed, antibodies response resulted higher in asymptomatic individuals, and although not statistically significant most probably due to the small sample size, the results from our sample suggest that there is a higher risk of symptoms and worse clinical course with lower (<1:80) nAb detected at diagnosis. Investigating the impact of the vaccination on the replication and potential infectivity, our data showed that the presence of nAb in serum at the time of diagnosis was not correlated with viral load in NPSs and did not preclude isolation of the replication-competent virus. According to other reports, vaccinated individuals during the initial phase of the infection may carry high viral loads (Ct ≤ 25) coupled with the presence of infectious virus in the upper respiratory tract with the potential of transmission (18, 20, 31, 32). Notably, the isolation rate and viral load according to the time elapsed from the first dose to diagnosis were found lower in samples collected from individuals who received the second vaccine dose as compared to the cases that resulted positive shortly after the vaccination (Group 1). In addition, the very small proportion of post-vaccine cases, together with recent studies on HCW cohorts with longitudinal analysis on breakthrough infections, highlighted the reduced transmission risk posed by vaccinated individuals (3, 14, 19, 28). It has been reported a lower risk of documented secondary cases in household members of vaccinated HCW and the more rapid decay of viral loads after diagnosis compared to unvaccinated patients, with a shorter duration of viral shedding and reasonably lower opportunity of contagiousness (19, 20, 33). The evaluation of the respiratory mucosal immune response in breakthrough infections cases may be of help to elucidate the mechanisms underlying transmission and diseases presentation in immunised individuals, as the antibodies in upper respiratory tract specimens may contribute to reducing the virus spread as well as limit the infection and the symptomaticity (34). The impact of emerging variants on the success of the vaccination campaigns is one of the main aspects which is continuously under investigation, as vaccine escape variants may be associated with increased vaccine failure (6). In the vaccinated individuals described in this study, the majority of infections were caused by Alpha variant (B.1.1.7), at that time considered as VOC, followed by the previously predominant strain in Lazio Region, B.1.177, and by the Gamma VOC (P.1). As previously reported in the United States (31), also in Lazio Region the temporal distribution of the variants identified in the vaccinated individuals clearly matches the pattern of strains circulation in the unvaccinated population during the same period, with no evidence of vaccine-related immune escape. This result may be influenced by the impact of the different prevalence of VOCs as compared to other scenarios, including the spread of the new predominant variant, Delta. For instance, Kustin et al. (35) reported that infection with Beta VOC in Israel was disproportionally detected in fully vaccinated individuals, while Alpha variant was disproportionally involved in infections diagnosed between 2 weeks after the first dose and 6 days after the second dose. The differences with our results may be due to different patterns of variants circulation, as, for example, circulation of Beta VOC was very tiny in our territory. According to other studies, the analysis of the Spike mutations in each lineage, broken down by Groups 1, 2, and 3, indicated that there is no selection for the enrichment of any particular mutation in fully vaccinated individuals (Group 3) as compared to individuals with incomplete vaccination (Groups 1 and 2) (36).

Our study presents some limitations that should be acknowledged. First, the study was a non-controlled observational study based on real-life data obtained from pandemic surveillance activities, aimed not to establish vaccine efficacy compared to a matched unvaccinated control group, but to report a virological characterisation of those patients reported with SARS-CoV-2 infection despite being vaccinated. Therefore, our observation should be replicated and extended on larger cohorts established *ad hoc*, also including other vaccine formulations as differences in incidence rates of breakthrough infections were observed in previous real-life studies (21, 26, 27). No follow-up samples were available for the post-vaccination infected individuals so that it was not possible to monitor viral loads dynamics and the shedding. Furthermore, the study was conducted before the emergence of the Delta variant which is now predominant worldwide and reported to be more transmissible with a higher risk of symptomatic infections (37). Finally, only three cases showed the presence of anti-N IgG suggesting seroconversion related to natural infection. nAb levels prior to infection were not available for most of the patients, therefore we cannot discriminate whether the titres obtained at the time of SARS-CoV-2 molecular test positivity were due to the vaccine response alone or elicited by the boost of the infection.

In conclusion, we observed that post-vaccine infections were mostly pauci/asymptomatic or mild, not associated with a failure in developing humoral response after vaccination. In addition, no preferential involvement in breakthrough infections of the variants with S mutations circulating during the study period emerged, as well as no enrichment in mutations following the vaccine.

Data collected worldwide highlight that vaccination represents a key factor to control morbidity and mortality of SARS-CoV-2 infection, as well as to reduce the public health burden of this pandemic and curb the social and economic global crisis. The evaluation of the immunological, virological, and clinical features behind vaccine breakthrough infections is an important aspect to investigate in order to better address prevention measures in the next phase of the COVID-19 pandemic.

## Data Availability Statement

The datasets presented in this study can be found in online repositories. The names of the repository/repositories and accession number(s) can be found in the article/Supplementary Material.

## Ethics Statement

No potentially identifiable human images or data are presented in the manuscript.

## Inmi Covid-19 Laboratory Surveillance Team

Abbate Isabella, Agrati Chiara, Agresta Alessandro, Aleo Loredana, Alonzi Tonino, Amendola Alessandra, Apollonio Claudia, Arduini Nicolina, Bartolini Barbara, Berno Giulia, Bettini Aurora, Biancone Silvia, Biava Mirella, Bibbò Angela, Bonfiglio Giulia, Bordi Licia, Brega Carla, Butera Ornella, Canali Marco, Cannas Angela, Capobianchi Maria Rosaria, Carletti Fabrizio, Carrara Stefania, Casetti Rita, Castilletti Concetta, Chiappini Roberta, Ciafrone Lucia, Cimini Eleonora, Coen Sabrina, Colavita Francesca, Condello Rossella, Coppola Antonio, D'Alessandro Gaetano, D'Arezzo Silvia, D'Amato Maurizio, De Santi Claudia, Di Caro Antonino, Di Filippo Stefania, De Giuli Chiara, Fabeni Lavinia, Federici Luigi, Felici Luisa, Ferraioli Valeria, Forbici Federica, Francalancia Massimo, Fusco Maria Concetta, Garbuglia Anna Rosa, Giombini Emanuela, Gramigna Giulia, Graziano Silvia, Gruber Cesare Ernesto Maria, Iazzetti Roberto, Khouri Daniele, Lalle Eleonora, Lapa Daniele, Leone Barbara, Leone Sara, Marafini Giovanni, Marchetti Federica, Massimino Chiara, Matusali Giulia, Mazzarelli Antonio, Meschi Silvia, Messina Francesco, Minosse Claudia, Montaldo Claudia, Mucciante Mirco, Nazzaro Clara, Neri Stefania, Nisii Carla, Petrivelli Elisabetta, Petroni Fabrizio, Petruccioli Elisa, Pisciotta Marina, Pitti Giovanni, Pizzi Daniele, Prota Gianluca, Rozera Gabriella, Rueca Martina, Sabatini Rossella, Santini Francesco, Sarti Silvia, Sberna Giuseppe, Sciamanna Roberta, Scionti Rachele, Selleri Marina, Specchiarello Eliana, Stellitano Chiara, Toffoletti Antonietta, Tonziello Gilda, Truffa Silvia, Turchi Federica, Valli Maria Beatrice, Vantaggio Valentina, Venditti Carolina, Vescovo Tiziana, Vincenti Donatella, Vulcano Antonella, Zambelli Emma.

## Author Contributions

CC, FCo, SM, BB, MC, and AD: study design. FCo, SM, MR, GBo, GM, DL, FCa, EL, LF, and Gbe: laboratory investigation. FCo, MR, EL, GBe, GBo, FV, MS, GD, and VP: data collection. FCa, FV, CG, EG, FM, and LF: data analysis. FCo, CG, CC, FV, MC, and AD: data interpretation. FCo and MC: writing, with revisions and comments from all authors. CC, AD, MC, and GI: funding acquisition and supervision. INMI COVID-19 Laboratory Surveillance Team: contributed to the data and samples collection, routine diagnostic, and epidemiological analyses. All authors contributed to the article and approved the submitted version.

## Funding

The study was performed in the framework of the SARS-CoV-2 surveillance and response program implemented by the Lazio Region Health Authority. This study was supported by funds to the Istituto Nazionale per le Malattie Infettive (INMI) Lazzaro Spallanzani IRCCS, Rome (Italy), from Ministero della Salute (Programma CCM 2020; Ricerca Corrente—linea 1; COVID-2020-12371817); the European Commission—Horizon 2020 (EU project 101003544—CoNVat; EU project 101005111-DECISION; EU project 101005075-KRONO) and the European Virus Archive—GLOBAL (Grant No. 871029).

## Conflict of Interest

The authors declare that the research was conducted in the absence of any commercial or financial relationships that could be construed as a potential conflict of interest.

## Publisher's Note

All claims expressed in this article are solely those of the authors and do not necessarily represent those of their affiliated organizations, or those of the publisher, the editors and the reviewers. Any product that may be evaluated in this article, or claim that may be made by its manufacturer, is not guaranteed or endorsed by the publisher.

## Supplementary Material

Supplementary Figure 1 Viral loads in NPSs and antibody response in sera collected in individuals tested positive at different times from the first dose vaccination. **(A)** Viral RNA levels are expressed as Ct of Orf1ab gene amplification, horizontal dashed lines represent the limit of detection of RT-PCR (Ct: 40). Samples yielding positive or negative viral culture are indicated in red and black, respectively; empty dots indicate samples for which viral culture was not performed. **(B)** Samples with available matching data on nAb titres and RNA viral load are shown according to the days from vaccination to testing (*n* = 44). nAb levels detected at the time of diagnosis are expressed as the reciprocal serum dilutions, horizontal lines represent starting dilution tested in MNT (1:10), samples below the line are considered not able to neutralise SARS-Cov-2. Samples from symptomatic and asymptomatic cases are indicated in red and black, respectively. The vertical dashed line represents the time of second dose vaccine administration.

Supplementary Figure 2 SARS-CoV-2 strains were detected in NPS samples collected from post-vaccination infections. **(A)** Percentages over a total of 63 sequences obtained are shown. Viral lineages detected in all vaccinated individuals (pie chart above) and divided according to the time elapsed from the first dose of vaccine to infection diagnosis (pie charts below) are indicated: Group 1 (time lapse 1–15 days), *n* = 9; Group 2 (16–30 days), *n* = 10; Group 3 (>30 days), *n* = 34; Unknown, vaccination date not available, *n* = 10. **(B)** Absolute frequencies of viral lineages were sequenced from the different vaccinated groups according to the time (month 2021) of infection diagnosis. VOI included B.1.525 lineage; Other includes strains belonging to B.1.1 lineage, B.1.1.39 lineage, B.1 lineage, and B.1.258.17 lineage.

Supplementary Figure 3 Amino acid substitutions were found in the Spike protein of SARS-CoV-2 sequences obtained from NGS analysis of vaccinated individuals. Mutations are shown according to the different vaccinated groups identified based on the time elapsed from the first dose of vaccine to testing: Group 1 (time lapse 1–15 days), *n* = 8; Group 2 (16–30 days), *n* = 10; Group 3 (>30 days), *n* = 30; VTU: Vaccination time unknown, *n* = 9. Mutations found in the receptor-binding domain (RBD) sequence are reported in light-red colour; mutations that cannot be confirmed nor excluded due to low coverage are reported in grey colour.

Supplementary Table S1 Factors associated with positive viral cultures on NPS collected from vaccinated individuals at the time of SARS-CoV-2 diagnosis.

Supplementary Table S2 Vaccinated individuals with available matching data on antibody and RNA viral load.

Supplementary Table S3 Neutralising antibodies against different SARS-CoV-2 variants.

## References

[1] Polack FP Thomas SJ Kitchin N Absalon J Gurtman A Lockhart S. Safety and Efficacy of the BNT162b2 mRNA Covid-19 Vaccine. N Engl J Med. (2020) 383:2603–15. 10.1056/NEJMoa2034577 33301246 PMC7745181

[2] Haas EJ Angulo FJ McLaughlin JM Anis E Singer SR Khan F. Impact and effectiveness of mRNA BNT162b2 vaccine against SARS-CoV-2 infections and COVID-19 cases, hospitalisations, and deaths following a nationwide vaccination campaign in Israel: an observational study using national surveillance data. Lancet. (2021) 397:1819–29. 10.1016/S0140-6736(21)00947-8 33964222 PMC8099315

[3] Dagan N Barda N Kepten E Miron O Perchik S Katz MA. BNT162b2 mRNA Covid-19 vaccine in a nationwide mass vaccination setting. N Engl J Med. (2021) 384:1412–23. 10.1056/NEJMoa2101765 33626250 PMC7944975

[4] Teran RA Walblay KA Shane EL Xydis S Gretsch S Gagner A. Postvaccination SARS-CoV-2 infections among skilled nursing facility residents and staff members—Chicago, Illinois, December 2020–March 2021. MMWR Morb Mortal Wkly Rep. (2021) 70:632–8. 10.15585/mmwr.mm7017e1 33914721 PMC8084122

[5] Stephenson J. COVID-19 Vaccinations in nursing home residents and staff give robust protection, though breakthrough infections still possible. JAMA Heal Forum. (2021) 2:e211195. 10.1001/jamahealthforum.2021.1195 36218817

[6] Hoffmann M Arora P Groß R Seidel A Hörnich BF Hahn AS. SARS-CoV-2 variants B.1.351 and P.1 escape from neutralizing antibodies. Cell. (2021) 184:2384–93.e12. 10.1016/j.cell.2021.03.036 33794143 PMC7980144

[7] Fontanet A Autran B Lina B Kieny MP Karim SSA Sridhar D. SARS-CoV-2 variants and ending the COVID-19 pandemic. Lancet. 2021 397:952–4. 10.1016/S0140-6736(21)00370-6 33581803 PMC7906631

[8] Wu K Werner AP Koch M Choi A Narayanan E Stewart-Jones GBE. Serum neutralizing activity elicited by mRNA-1273 vaccine. N Engl J Med. (2021) 384:1468–70. 10.1056/NEJMc2102179 33730471 PMC8008744

[9] Rueca M Giombini E Messina F Bartolini B Di Caro A Capobianchi MR. ESCA pipeline: Easy-to-use SARS-CoV-2 genome Assembler. bioRxiv. (2021) Jan 1 2021.05.21.445156. Available from: http://biorxiv.org/content/early/2021/05/21/2021.05.21.445156.abstract

[10] Shepard SS Meno S Bahl J Wilson MM Barnes J Neuhaus E. Viral deep sequencing needs an adaptive approach: IRMA, the iterative refinement meta-assembler. BMC Genomics. (2016) 17:708. 10.1186/s12864-016-3030-6 27595578 PMC5011931

[11] Elbe S Buckland-Merrett G. Data, disease and diplomacy: GISAID's innovative contribution to global health. Glob challenges. (2017) 1:33–46. 10.1002/gch2.1018 31565258 PMC6607375

[12] Colavita F Lapa D Carletti F Lalle E Messina F Rueca M. Virological characterization of the first 2 COVID-19 patients diagnosed in italy: phylogenetic analysis, virus shedding profile from different body sites, and antibody response kinetics. Open Forum Infect Dis. (2020) 7:ofaa403. 10.1093/ofid/ofaa403/5900725 33527081 PMC7499768

[13] Matusali G Colavita F Lapa D Meschi S Bordi L Piselli P. SARS-CoV-2 serum neutralization assay: a traditional tool for a brand-new virus. Viruses. (2021) 13:655. 10.3390/v13040655 33920222 PMC8069482

[14] Levine-Tiefenbrun M Yelin I Katz R Herzel E Golan Z Schreiber L. Initial report of decreased SARS-CoV-2 viral load after inoculation with the BNT162b2 vaccine. Nat Med. (2021) 27:790–2. 10.1038/s41591-021-01316-7 33782619

[15] Hall VJ Foulkes S Charlett A Atti A Monk EJ Simmons R. Do antibody positive healthcare workers have lower SARS-CoV-2 infection rates than antibody negative healthcare workers? Large Multi-Centre Prospective Cohort Study (The SIREN Study), England: June to November 2020. medRxiv. (2021). 10.1101/2021.01.13.2124964234722359

[16] Keehner J Horton LE Pfeffer MA Longhurst CA Schooley RT Currier JS. SARS-CoV-2 infection after vaccination in health care workers in California. N Engl J Med. (2021) 384:1774–5. 10.1056/NEJMc2101927 33755376 PMC8008750

[17] Thompson MG Burgess JL Naleway AL Tyner HL Yoon SK Meece J. Interim estimates of vaccine effectiveness of BNT162b2 and mRNA-1273 COVID-19 vaccines in preventing SARS-CoV-2 infection among health care personnel, first responders, and other essential and frontline workers—Eight U.S. Locations, December 2020-March. MMWR Morb Mortal Wkly Rep. (2021) 70:495–500. 10.15585/mmwr.mm7013e3 33793460 PMC8022879

[18] Klompas M. Understanding breakthrough infections following mRNA SARS-CoV-2 vaccination. JAMA. (2021) 326:2018–20. 10.1001/jama.2021.19063 34734985

[19] Rovida F Cassaniti I Paolucci S Percivalle E Sarasini A Piralla A. SARS-CoV-2 vaccine breakthrough infections with the alpha variant are asymptomatic or mildly symptomatic among health care workers. Nat Commun. (2021) 12:6032. 10.1038/s41467-021-26154-6 34654808 PMC8521593

[20] Chia PY Ong SWX Chiew CJ Ang LW Chavatte J-M Mak T-M. Virological and serological kinetics of SARS-CoV-2 Delta variant vaccine-breakthrough infections: a multi-center cohort study. medRxiv. (2021) 2021.07.28.21261295. Available from: https://www.medrxiv.org/content/10.1101/2021.07.28.21261295v1%0Ahttps://www.medrxiv.org/content/10.1101/2021.07.28.21261295v1.abstract 10.1016/j.cmi.2021.11.010 PMC8608661 34826623

[21] Cong Liu Junghwan Lee Casey Ta Ali Soroush James R. Rogers Jae Hyun Kim Karthik Natarajan Jason Zucker CW. A Retrospective Analysis of COVID-19 mRNA vaccine breakthrough infections—risk factors and vaccine effectiveness. medRxiv. (2021).34642696

[22] Levin EG Lustig Y Cohen C Fluss R Indenbaum V Amit S. Waning immune humoral response to BNT162b2 Covid-19 vaccine over 6 months. N Engl J Med. (2021) 385:e84. 10.1056/NEJMoa2114583 34614326 PMC8522797

[23] Sanyaolu A Okorie C Marinkovic A Patidar R Younis K Desai P. Comorbidity and its Impact on Patients with COVID-19. SN Compr Clin Med. (2020) 25:1–8. 10.1007/s42399-020-00363-4 32838147 PMC7314621

[24] Post N Eddy D Huntley C van Schalkwyk MCI Shrotri M Leeman D. Antibody response to SARS-CoV-2 infection in humans: a systematic review. PLoS ONE. (2020) 15:e0244126. 10.1371/journal.pone.0244126 33382764 PMC7775097

[25] Hacisuleyman E Hale C Saito Y Blachere NE Bergh M Conlon EG. Vaccine Breakthrough Infections with SARS-CoV-2 Variants. N Engl J Med. (2021) 384:2212–8. 10.1056/NEJMoa2105000 33882219 PMC8117968

[26] Juthani P V Gupta A Borges KA Price CC Lee AI Won CH. Hospitalisation among vaccine breakthrough COVID-19 infections. Lancet Infect Dis. (2021) 21:1485–6. 10.1016/S1473-3099(21)00558-2 34506735 PMC8423430

[27] Bosch W Cowart JB Bhakta S Carter RE Wadei HM Shah SZ. Coronavirus disease 2019 vaccine-breakthrough infections requiring hospitalization in mayo clinic florida through August 2021. Clin Infect Dis. (2021) ciab932. 10.1093/cid/ciab932 34726700 PMC8689905

[28] Bergwerk M Gonen T Lustig Y Amit S Lipsitch M Cohen C. Covid-19 breakthrough infections in vaccinated health care workers. N Engl J Med. (2021) 385:1474–84. 10.1056/NEJMoa2109072 34320281 PMC8362591

[29] Brosh-Nissimov T Orenbuch-Harroch E Chowers M Elbaz M Nesher L Stein M. BNT162b2 vaccine breakthrough: clinical characteristics of 152 fully vaccinated hospitalized COVID-19 patients in Israel. Clin Microbiol Infect. (2021) 27:1652–7. 10.1016/j.cmi.2021.06.036 34245907 PMC8261136

[30] Novazzi F Taborelli S Baj A Focosi D Maggi F. Asymptomatic SARS-CoV-2 vaccine breakthrough infections in health care workers identified through routine universal surveillance testing. Ann Intern Med. (2021) 174:1770–2. 10.7326/M21-3486 34662153 PMC8524618

[31] Birhane M Bressler S Chang G Clark T Dorough L Fischer M. COVID-19 vaccine breakthrough infections reported to CDC—United States, January 1–April 30, 2021. MMWR Morb Mortal Wkly Rep. (2021) 70:792–3. 10.15585/mmwr.mm7021e3 34043615 PMC8158893

[32] Jung J Sung H KS. Covid-19 Breakthrough infections in vaccinated health care workers. N Engl J Med. (2021) 385:1629–31. 10.1056/NEJMc2113497 34587378

[33] Shamier MC Tostmann A Bogers S Wilde J de IJpelaar J Kleij WA van der. Virological characteristics of SARS-CoV-2 vaccine breakthrough infections in health care workers. medRxiv. (2021) 2021.08.20.21262158. Available from: https://www.medrxiv.org/content/10.1101/2021.08.20.21262158v1%0Ahttps://www.medrxiv.org/content/10.1101/2021.08.20.21262158v1.abstract

[34] Mostaghimi D Valdez CN Larson HT Kalinich CC Iwasaki A. Prevention of host-to-host transmission by SARS-CoV-2 vaccines. Lancet Infect Dis. (2021) S1473-3099(21)00472-2. 10.1016/S1473-3099(21)00472-2 34534512 PMC8439617

[35] Kustin T Harel N Finkel U Perchik S Harari S Tahor M. Evidence for increased breakthrough rates of SARS-CoV-2 variants of concern in BNT162b2-mRNA-vaccinated individuals. Nat Med. (2021) 27:1379–84. 10.1038/s41591-021-01413-7 34127854 PMC8363499

[36] Baltas I Boshier FAT Williams CA Bayzid N Cotic M Afonso Guerra-Assunção J. Post-vaccination coronavirus disease 2019: a case-control study and genomic analysis of 119 breakthrough infections in partially vaccinated individuals. Clin Infect Dis. (2021). 10.1093/cid/ciab714 34410361 PMC8513403

[37] Luo CH Morris CP Sachithanandham J Amadi A Gaston D Li M. Infection with the SARS-CoV-2 delta variant is associated with higher infectious virus loads compared to the alpha variant in both unvaccinated and vaccinated individuals. medRxiv Prepr Serv Heal Sci. (2021) ciab986. 10.1101/2021.08.15.21262077 34462756 PMC8404894

