# Supplementary figures and images for: Virological and Serological Characterisation of SARS-CoV-2 Infections Diagnosed After mRNA BNT162b2 Vaccination Between December 2020 and March 2021
Source: Front Med (Lausanne). 2022 Jan 20;8:815870. doi: 10.3389/fmed.2021.815870 (PMC8810639; doi:10.3389/fmed.2021.815870)

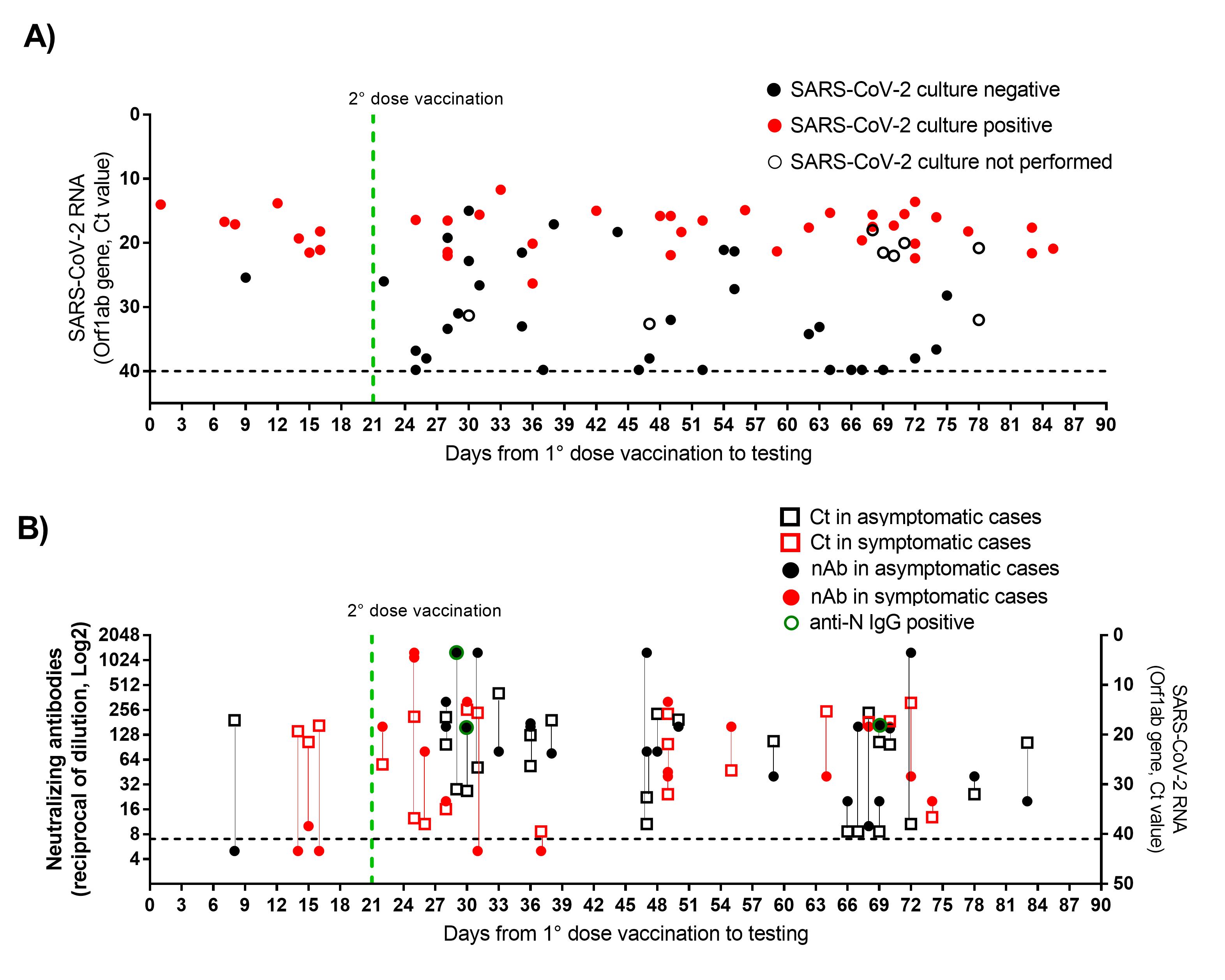

Supplement: Supplementary Figure 1 — Viral loads in NPSs and antibody response in sera collected in individuals tested positive at different times from the first dose vaccination. (A) Viral RNA levels are expressed as Ct of Orf1ab gene amplification, horizontal dashed lines represent the limit of detection of RT-PCR (Ct: 40). Samples yielding positive or negative viral culture are indicated in red and black, respectively; empty dots indicate samples for which viral culture was not performed. (B) Samples with available matching data on nAb titres and RNA viral load are shown according to the days from vaccination to testing (n = 44). nAb levels detected at the time of diagnosis are expressed as the reciprocal serum dilutions, horizontal lines represent starting dilution tested in MNT (1:10), samples below the line are considered not able to neutralise SARS-Cov-2. Samples from symptomatic and asymptomatic cases are indicated in red and black, respectively. The vertical dashed line represents the time of second dose vaccine administration. [file Image_1.JPEG]

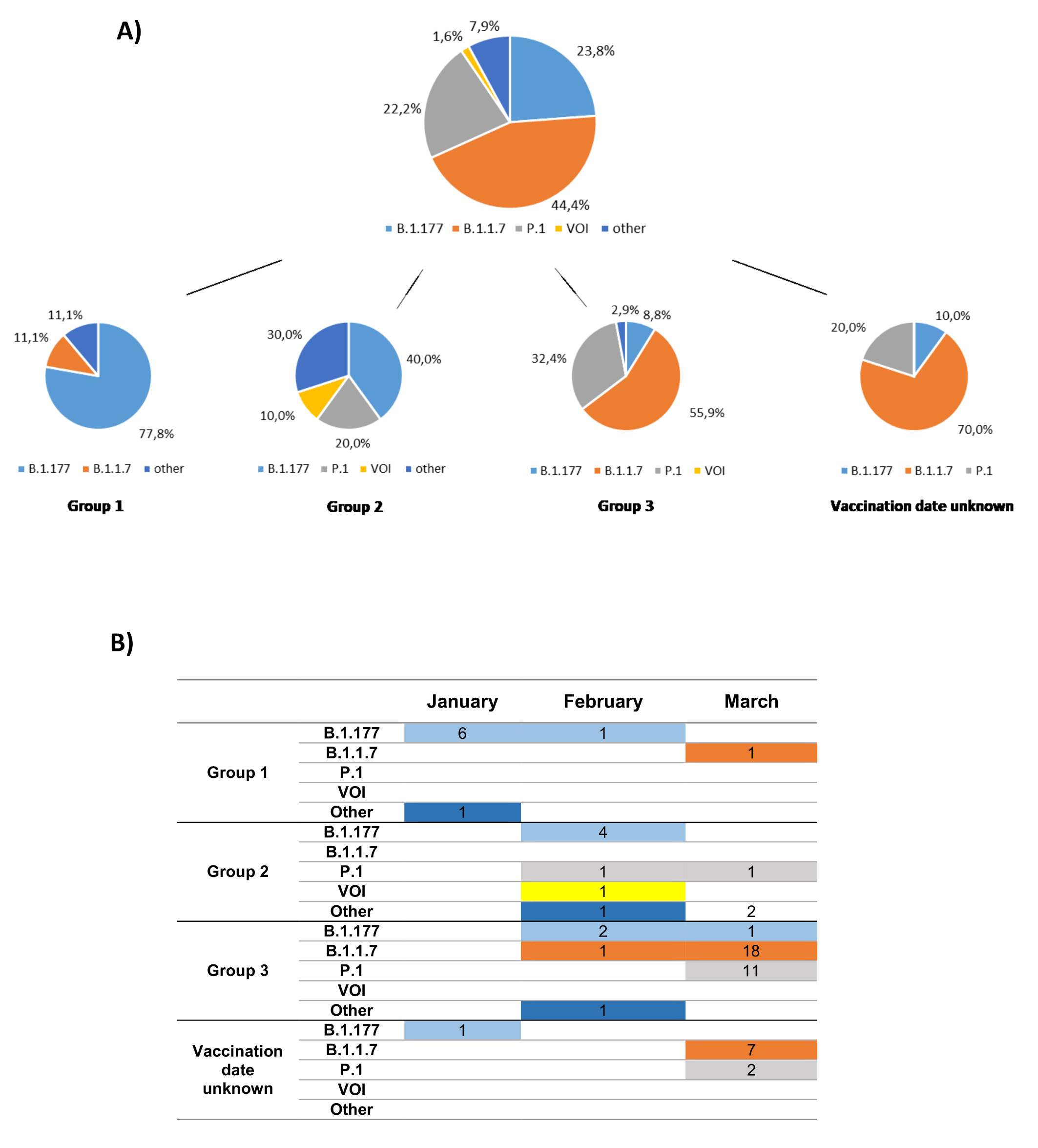

Supplement: Supplementary Figure 2 — SARS-CoV-2 strains were detected in NPS samples collected from post-vaccination infections. (A) Percentages over a total of 63 sequences obtained are shown. Viral lineages detected in all vaccinated individuals (pie chart above) and divided according to the time elapsed from the first dose of vaccine to infection diagnosis (pie charts below) are indicated: Group 1 (time lapse 1–15 days), n = 9; Group 2 (16–30 days), n = 10; Group 3 (>30 days), n = 34; Unknown, vaccination date not available, n = 10. (B) Absolute frequencies of viral lineages were sequenced from the different vaccinated groups according to the time (month 2021) of infection diagnosis. VOI included B.1.525 lineage; Other includes strains belonging to B.1.1 lineage, B.1.1.39 lineage, B.1 lineage, and B.1.258.17 lineage. [file Image_2.JPEG]

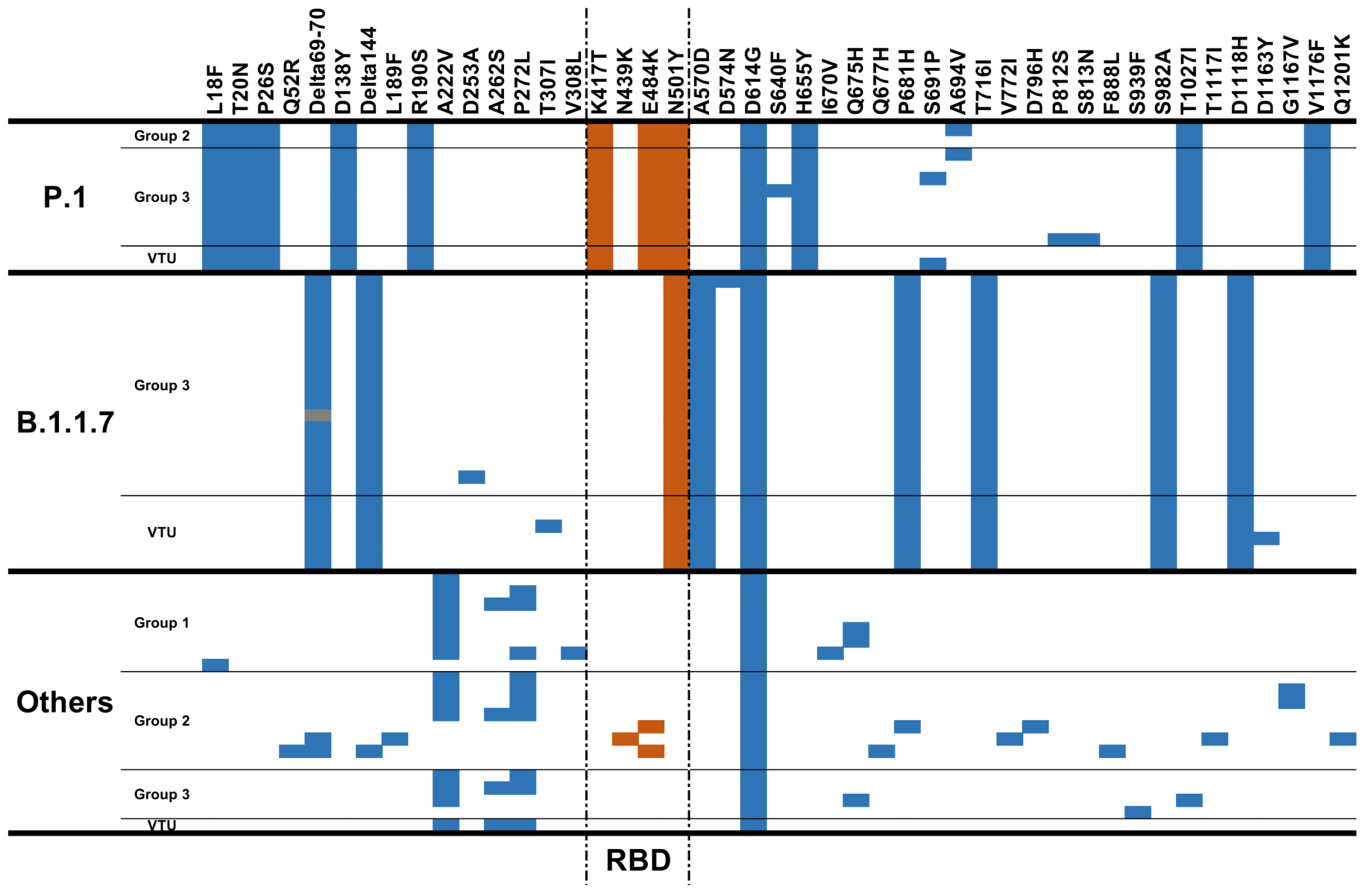

Supplement: Supplementary Figure 3 — Amino acid substitutions were found in the Spike protein of SARS-CoV-2 sequences obtained from NGS analysis of vaccinated individuals. Mutations are shown according to the different vaccinated groups identified based on the time elapsed from the first dose of vaccine to testing: Group 1 (time lapse 1–15 days), n = 8; Group 2 (16–30 days), n = 10; Group 3 (>30 days), n = 30; VTU: Vaccination time unknown, n = 9. Mutations found in the receptor-binding domain (RBD) sequence are reported in light-red colour; mutations that cannot be confirmed nor excluded due to low coverage are reported in grey colour. [file Image_3.JPEG]
