# Supplementary material for: Virological and Serological Characterisation of SARS-CoV-2 Infections Diagnosed After mRNA BNT162b2 Vaccination Between December 2020 and March 2021
Source: Front Med (Lausanne). 2022 Jan 20;8:815870. doi: 10.3389/fmed.2021.815870 (PMC8810639; doi:10.3389/fmed.2021.815870)
Supplement: Supplementary Table S1 — Factors associated with positive viral cultures on NPS collected from vaccinated individuals at the time of SARS-CoV-2 diagnosis. [file Table_1.DOCX]

**Table S1. Factors associated with positive viral cultures on NPS collected from vaccinated individuals at the time of SARS-CoV-2 diagnosis.**

| **Variable** | **Positive viral culture**  **n (%)** | **Univariate analysis**  **OR (95% CI)** | **p-value** |
| --- | --- | --- | --- |
| RT-PCR, Orf1ab gene (N=84) | | | |
| *Ct value≤25^§^ (n=55)* | 43 (78.2%) | 100.30 (12.35-815.07) | **<0.001** |
| *Ct value>25 (n=29)* | 1 (3.4%) |  |  |
| Time of the infection diagnosis (N=72) | | | |
| *10 days after the second dose^§^ (n=49)* | 27 (55.1%) | 1.02 (0.37-2.80) | 0.966 |
| *10 days before the second dose (n=23)* | 17 (73.9%) |  |  |
| Presence of symptoms at diagnosis (N=84) |  |  |  |
| *Yes^§^ (n=44)* | 24 (54.4%) | 1.20 (0.51-2.83) | 0.677 |
| *No (n=40)* | 20 (50.0%) |  |  |
| Presence of P.1 (Gamma, N=57) |  |  |  |
| *Yes^§^ (n=14)* | 10 (71.4 %) | 0.97 (0.25-3.69) | 0.962 |
| *No (n=43)* | 31 (72.1%) |  |  |
| Presence of B.1.1.7 (Alpha, N=57) |  |  |  |
| *Yes^§^ (n=23)* | 16 (69.6%) | 0.82 (0.26-2.65) | 0.744 |
| *No (n=34)* | 26 (76.5%) |  |  |
| Presence of nAb in serum (N=44) |  |  |  |
| *Yes^§^ (n=37)* | 19 (51.3%) | 0.18 (0.02-1.61) | 0.124 |
| *No (n=7)* | 6 (85.7%) |  |  |
| nAb titres in serum (N=44) |  |  |  |
| *≥1:80^§^ (n=25)* | 10 (40.0%) | 0.28 (0.08-1.02) | 0.054 |
| *<1:80 (n=19)* | 14 (73.7%) |  |  |

Abbreviation: OR, Odds ratio; CI, confidence interval; N, total data available for the analysis; n, number of data. Boldface indicates factor significantly associated (p<0.05) with positive viral cultures.

^§^ Reference group (dummy)
