# Supplementary material for: Virological and Serological Characterisation of SARS-CoV-2 Infections Diagnosed After mRNA BNT162b2 Vaccination Between December 2020 and March 2021
Source: Front Med (Lausanne). 2022 Jan 20;8:815870. doi: 10.3389/fmed.2021.815870 (PMC8810639; doi:10.3389/fmed.2021.815870)
Supplement: Supplementary Table S2 — Vaccinated individuals with available matching data on antibody and RNA viral load. [file Table_2.docx]

**Table S2. Vaccinated individuals with available matching data on antibody and RNA viral load.**

| **ID** | **Viral load (Ct values)** | **Viral culture** | **Viral Strain** | **anti-S IgG (BAU/mL≥7.1)** | **anti-N IgG (Index≥1.4)** | **nAb (VNT90≥1:10)** | **DTV** | **DSV** |
| --- | --- | --- | --- | --- | --- | --- | --- | --- |
| 1 | 17.1 | Positive | B.1.177 | <7.1 | 0.02 | <1:10 | 8 | n.a. |
| 2 | 19.3 | Positive | B.1.177 | 58.2 | 0.02 | <1:10 | 14 | 14 |
| 3 | 21.5 | Positive | B.1.177 | 25.9 | 0.02 | 1:10 | 15 | 12 |
| 4 | 18.2 | Positive | B.1.177 | 1130.6 | 0.02 | <1:10 | 16 | 13 |
| 5 | 26.0 | Negative | B.1.177 | 5549.1 | 0.56 | 1:160 | 22 | 22 |
| 6 | 16.4 | Positive | B.1.525 | 11360.0 | 0.16 | 1:1280 | 25 | 21 |
| 7 | 36.8 | Negative | n.p. | 11360.0 | 1.08 | 1:1280 | 25 | 21 |
| 8 | 38.0 | Negative | n.p. | 1615.2 | 0.11 | 1:80 | 26 | 24 |
| 9 | 33.4 | Negative | n.p. | 733.2 | 0.03 | 1:20 | 28 | 24 |
| 10 | 22.0 | Positive | B.1.177 | 5306.6 | 0.02 | 1:160 | 28 | n.a. |
| 11 | 16.5 | Positive | P.1 | 2.284.60 | 0.56 | 1:320 | 28 | n.a. |
| 12 | 31.0 | Negative | n.p. | 2678.0 | **6.47** | 1:1280 | 29 | n.a. |
| 13 | 31.3 | n.p. | n.p. | 1174.1 | **4.35** | 1:160 | 30 | n.a. |
| 14 | 15.0 | Negative | P.1 | 9889.7 | 0.05 | 1:320 | 30 | 27 |
| 15 | 15.6 | Positive | B.1.1.7 | 17.1 | 0.02 | <1:10 | 31 | 31 |
| 16 | 26.6 | Negative | B.1.177 | 4397.2 | 0.81 | 1:1280 | 31 | n.a. |
| 17 | 11.7 | Positive | B.1.177 | 3311.7 | 0.11 | 1:80 | 33 | n.a. |
| 18 | 26.3 | Positive | B.1.177 | 208.8 | 0.01 | 1:160 | 36 | n.a. |
| 19 | 20.1 | Positive | B.1.1.39 | 1632.8 | 0.03 | 1:160 | 36 | n.a. |
| 20 | 39.0 | Negative | B.1.1.7 | <7.1 | 0.02 | <1:10 | 37 | 37 |
| 21 | 17.1 | Negative | P.1 | 799.1 | 0.19 | 1:80 | 38 | n.a. |
| 22 | 38.0 | Negative | n.p. | 313.7 | 0.17 | 1:1280 | 47 | n.a. |
| 23 | 32.6 | n.p. | n.p. | 2453.0 | 0.07 | 1:80 | 47 | n.a. |
| 24 | 15.8 | Positive | n.p. | 950.8 | 0.01 | 1:80 | 48 | n.a. |
| 25 | 21.9 | Positive | n.p. | 467.3 | 0.05 | 1:40 | 49 | 45 |
| 26 | 15.8 | Positive | P.1 | 861.7 | 0.07 | 1:320 | 49 | 46 |
| 27 | 32.0 | Negative | n.p. | 568.2 | 0.04 | 1:40 | 49 | 47 |
| 28 | 18.3 | Positive | B.1.1.7 | 2179.8 | 0.04 | 1:160 | 50 | n.a. |
| 29 | 27.2 | Negative | n.p. | 825.7 | 0.17 | 1:160 | 55 | 53 |
| 30 | 21.3 | Positive | B.1.1.7 | 575.0 | 0.08 | 1:40 | 59 | n.a. |
| 31 | 15.3 | Positive | B.1.177 | 200.8 | 0.03 | 1:40 | 64 | 57 |
| 32 | 38.0 | Negative | n.p. | 529.8 | 0.01 | 1:20 | 66 | n.a. |
| 33 | 38.0 | Negative | n.p. | 1379.6 | 0.06 | 1:160 | 67 | n.a. |
| 34 | 15.6 | Positive | B.1.1.7 | 70.2 | 0.02 | 1:10 | 68 | n.a. |
| 35 | 17.5 | Positive | B.1.1.7 | 599.1 | 0.05 | 1:160 | 68 | 64 |
| 36 | 37.0 | Negative | n.p. | 1742.9 | **2.55** | 1:160 | 69 | n.a. |
| 37 | 21.5 | n.p. | B.1.1.7 | 475.0 | 0.04 | 1:20 | 69 | n.a. |
| (continued next page) | | | | | | | | |
| 38 | 17.3 | Positive | B.1.1.7 | 208.8 | 0.01 | 1:160 | 70 | 67 |
| 39 | 22.0 | n.p. | P.1 | 1903.1 | 0.02 | 1:160 | 70 | n.a. |
| 40 | 13.6 | Positive | P.1 | 574.7 | 0.03 | 1:40 | 72 | 70 |
| 41 | 38.0 | Negative | n.p. | 3569.7 | 0.49 | 1:1280 | 72 | n.a. |
| 42 | 36.6 | Negative | n.p. | 597.9 | 0.1 | 1:20 | 74 | 73 |
| 43 | 32.0 | n.p. | n.p. | 481.2 | 0.54 | 1:40 | 78 | n.a. |
| 44 | 21.6 | Positive | B.1.1.7 | 506.8 | 0.01 | 1:20 | 83 | n.a. |
| 45 | 20.0 | Negative | P.1 | 676.6 | 0.02 | 1:80 | n.a. | n.a. |
| 46 | 18.9 | n.p. | B.1.1.7 | 69.6 | 0.03 | <1:10 | n.a. | n.a. |
| 47 | 16.3 | Positive | P.1 | 732.6 | 0.02 | 1:40 | n.a. | n.a. |
| 48 | 14.2 | Positive | B.1.1.7 | 382.6 | 0.02 | <1:10 | n.a. | n.a. |
| 49 | 33.1 | Negative | n.p. | 609.5 | 0.3 | 1:80 | n.a. | n.a. |
| 50 | 12.1 | Positive | B.1.1.7 | 36.3 | 0.02 | <1:10 | n.a. | n.a. |

Abbreviation: DTV, days from first dose vaccination to testing; DSV, days from first dose vaccination to symptoms onset (for symptomatic individuals only); n.p., not performed; n.a., not available, as vaccination date unknown (DTV), or absence of symptoms at diagnosis (DSV).
