# Supplementary material for: Virological and Serological Characterisation of SARS-CoV-2 Infections Diagnosed After mRNA BNT162b2 Vaccination Between December 2020 and March 2021
Source: Front Med (Lausanne). 2022 Jan 20;8:815870. doi: 10.3389/fmed.2021.815870 (PMC8810639; doi:10.3389/fmed.2021.815870)
Supplement: Supplementary Table S3 — Neutralising antibodies against different SARS-CoV-2 variants. [file Table_3.DOCX]

**Table S3. Neutralizing antibodies against different SARS-CoV-2 variants.**

| SARS-CoV-2 strain causing infection | nAb titre  against  G (D614G) | nAb titre against  GR - P.1 | nAb titre  against  GRY – B.1.1.7 |
| --- | --- | --- | --- |
| GV - B.1.177 | 1:160 | 1:320 | 1:320 |
|  | 1:80 | 1:320 | 1:320 |
|  | 1:40 | 1:160 | 1:80 |
|  | 1:160 | 1:320 | 1:160 |
|  | 1:160 | 1:160 | 1:160 |
|  | 1:40 | 1:10 | 1:10 |
| GR – P.1  (Gamma) | 1:320 | 1:640 | 1:640 |
|  | 1:40 | 1:40 | 1:20 |
|  | 1:80 | 1:80 | 1:40 |
|  | 1:320 | 1:160 | 1:80 |
|  | 1:40 | 1:80 | 1:40 |
|  | 1:160 | 1:640 | 1:640 |
|  | 1:320 | 1:640 | 1:160 |
| GRY – B.1.1.7  (Alpha) | 1:160 | 1:80 | 1:80 |
|  | 1:20 | <1:10 | 1:10 |
|  | 1:160 | 1:20 | 1:20 |
|  | 1:20 | 1:40 | 1:20 |
|  | 1:40 | 1:10 | 1:20 |

nAb titre<1:10 is considered as negative.

Friedman-Dunn test, p=0.656
